# European Society of Radiology (ESR) and American College of Radiology (ACR) report of the 2015 global summit on radiological quality and safety

**DOI:** 10.1007/s13244-016-0493-6

**Published:** 2016-05-19

**Authors:** European Society of Radiology (ESR), American College of Radiology (ACR)

**Affiliations:** European Society of Radiology (ESR), Neutorgasse 9/2, 1010 Vienna, Austria; American College of Radiology, 1891 Preston White Dr., Reston, VA 20191 USA

**Keywords:** Quality, Safety, Practice, Performance, Value

## Abstract

**Abstract:**

The American College of Radiology (ACR) and the European Society of Radiology (ESR) held the second joint Global Summit on Radiological Quality and Safety on October 10–11, 2015 in Barcelona. The programme addressed the issues of safety, professional performance, practice improvement and customer service. Participants came from national and international radiological societies; partner medical societies; global organisations such as the International Atomic Energy Agency and the World Health Organisation; and patient advocacy groups. The objective was to exchange ideas and develop common strategies to improve and harmonise quality and safety in radiology on a global level. Participants debated and proposed improvement initiatives at the conclusion of the meeting.

***Main Messages*:**

• *Radiologists must adapt to demonstrate their value to the healthcare system.*

• *Integration of quality and safety policies is crucial for our profession.*

• *Excellent patient care includes good communication and direct involvement in clinical problem solving.*

• *Culture is shifting towards clinical decision support tools for appropriate use of imaging.*

• *“Big data” is a great opportunity for radiologists to improve the quality of patient care.*

## Introduction

Constantly evolving technologies and economic challenges make it imperative for radiologists to adapt to rapidly changing environments, and to demonstrate their value to patients and the healthcare system. Customer satisfaction and professional performance are key indicators, but require appropriate metrics for measurement. The precise challenges for radiology may vary worldwide, but on a global level include the need to balance economic efficiency and 24-hour coverage with the need to ensure patient safety and maintain high quality. These issues affect us all and benefit from a global strategic approach.

### Addressing the challenges of changes to the provision of radiology

Teleradiology is one of the fastest growing sectors in healthcare. This is partly driven by the challenges of providing 24-hour care in the face of insufficient numbers of radiologists worldwide. Many radiologists fear that outsourcing will lead to increasing commoditisation and cost competition, to the detriment of quality; and alternative models, such as conglomerates or local networks (insourcing) are increasingly being explored. Radiologists must ensure that where it is used, outsourcing provides additional value without sacrificing quality, but how best to evaluate teleradiology services is not yet established.

Both the American College of Radiology (ACR) and the European Society of Radiology (ESR) published white papers on teleradiology in 2014, and the ESR has recently carried out surveys^1^ on attitudes to teleradiology, which will help to shape future policy. There are concerns about outsourcing in particular, and the impact that teleradiology may have on the future shape of the specialty, and role of the radiologist.

### 24-hour coverage

24-hour coverage traditionally involved emergency cases only, but subspecialised services are increasingly expected to be available around the clock. The trend is for hybrid models of in-sourced and out-sourced services, and for staff radiologists to work from home or in different time zones.

The quality of service may be influenced by shift length, so study volume should be carefully considered. Adverse health effects of long shifts include sleep deprivation, insomnia, drug abuse, depression, cardiovascular diseases, cognitive deficits and impaired performance. These effects can lead to lower job satisfaction and motivation, and affect work quality, general performance and ultimately patient care.

### Public and patient perception of radiologists

In order for radiologists to change the way they are perceived they will have to establish their value, not just as report generators, but as guardians of patient safety through justification and limitation (optimisation) of radiation exposure, and efficient management of diagnostic and therapeutic imaging pathways. It is imperative to establish the value of the radiologists to patient care through good communication with both patients and referrers, and via more direct involvement in clinical problem-solving and patient care. Patient groups expressed the view that direct discussion of imaging investigations, not just a written report, would be valued.

### Measuring quality

Quality means different things to different people. Patients expect to receive high-quality examinations and reports in a timely and efficient manner, and to be assured that their radiation exposure is minimised and safety of procedures assured. Payers have a particular interest in appropriateness, and good outcomes with minimisation of costs.

In the near future in the USA, healthcare payments will be increasingly linked to quality and outcome metrics. Examples of good practice include department quality and value scorecards to capture data on appropriateness, and various quality metrics to provide evidence-based data. However, the metrics radiologists currently look at only measure processes. It is in the interests of radiologists themselves to demonstrate the value of their service, and develop appropriate metrics, which capture their true contribution and added value to patient care. Our industry partners may be helpful in developing such tools.

Research output, teaching activities, and multidisciplinary conference support are measurable. Much more difficult to measure are contribution to team performance and what difference the radiologist makes to patient outcome, although some of this broader contribution can be captured in 360-degree appraisal questionnaires.

Using a scorecard that captures quantified value-added actions performed by radiologists like the radiology value-added matrix (http://www.acr.org/∼/media/ACR/Images/Advocacy/Imaging%203/Case%20Studies/The%20Value%20of%20Hard%20Work/slide%201.jpg) could help show administrators that radiologists are moving towards a team-based approach that could positively impact patient care.

### Professional performance

At any one time, 1 to 6 % of doctors will be in some way be underperforming. As many as 5 % may demonstrate disruptive behaviour; 1 in 17 drinks alcohol to excess or takes drugs at some time in their professional lives; and 15 % have their performance affected by illness sometime in their life. Programmes exist to improve personal interaction skills in the workplace and to assist in case of mental illness and alcohol or substance abuse. As a group, late-career radiologists are now finding career support on topics such as health issues and how to plan for retirement.

Regular local appraisal may also pick up professional performance issues at an early stage. Maintenance of the certification programme of the American Board of Radiology compiles competencies and credentials, and expects people to demonstrate life-learning assessment and participate in practical quality improvement efforts. Performance can also be assessed through peer review and multiple source feedback, or by using systems like ACR’s Radpeer. The panel strongly agreed that current metrics such as peer review had major limitations, and that there are substantial improvement opportunities in this area.

### Audits in radiology

Audits are inextricably linked with quality, but suffer from a poor reputation. For professionals to spend time on audits, the process must be relevant and be perceived as having inherent value, for example, linking it to certification and/or reimbursement. Linking participation in registries with certification is a strategy that the American Board of Radiology is currently exploring.

### Customer service

On the requesting provider side of imaging, there is a cultural shift towards clinical decision support (CDS) tools for the appropriate use of imaging investigations. Radiologists must be fully engaged throughout the entire process — from the initial imaging request, to exam protocoling, image interpretation and reporting, and actionable recommendations.

An example is ACR Select, a comprehensive national standards CDS database that provides evidence-based decision support for the appropriate use of all medical imaging procedures, which has been adopted by over 100 facilities and handles 20 million orders per year. The ESR is developing together with the ACR generic, evidence-based guidelines for use in a CDS system (ESRiGuide) with flexibility for localisation following the model of ACR Select based on an agreement with ACR.

In Europe, the EURATOM basic safety standards (BSS) directive goes into effect in 2018 and mandates guideline availability in all member states (http://eur-lex.europa.eu/LexUriServ/LexUriServ.do?uri=OJ:L:2014:013:0001:0073:EN:PDF). However, a survey (http://www.myesr.org/cms/website.php?id=/en/eu_affairs/newfilename.htm) done by the ESR showed that referral guidelines, although available since 1997, are rarely used in the EU countries, and that the situation is heterogeneous between member states.

On the reporting side of imaging, studies have shown that a large majority of referring practitioners prefer structured reports (SR) to free text reports [1, 2]^2^,^3^, although the level of enthusiasm amongst radiologists has usually lagged behind. In addition, in a recent survey, the ACR found that there was strong interest for radiology reports to contain evidence-based guidelines and recommendations. Information technology (IT) tools should be developed so guidelines are readily available, for ease of use.

### Malpractice

A survey conducted by the Journal of the American College of Radiology in 2012 revealed that nearly half of radiologists in the USA had either been defendants or had negligence claims made against them [3]^4^. In 2014, over 3.5bn USD were spent in settlements and pay-outs for medical malpractice. In Europe, there is some variation, but in many countries, legal costs are increasing. An Italian report published in 2009 showed that 15,000 legal actions were taken against physicians and that over 10bn EUR were spend in compensation per year [4]^5^.

There has been increasing interest in open disclosure of error, with formal apology to the patient, a practice which has been shown to decrease the number of legal claims [5]^6^. Patient advocate representatives reminded the panel that their response to error may affect how patients react, suggesting patients are more likely to sue if they feel the organisation or individual is trying to conceal error or is using delaying tactics.

### Professional shortages

A shortage of radiologists and radiographers is a problem in many parts of the world. Where there is uneven distribution of the workforce, this impacts patient access to imaging. The increasing demand for imaging has also strained resources over the past decades.

Initiatives aimed at mixing skills have helped meet the demand but radiology must become more attractive as a specialty and radiographers must improve their skills to act as gatekeepers to access to imaging equipment.

In times of professional shortage, mobility of the workforce is important. Mobility of radiologists in the EU brings benefits but the diversity and disparity in language, training and practice between countries can result in a number of problems.

The ESR has supported the mobility of young graduates by introducing the concept of a European curriculum and putting emphasis on continuing life-long medical education. It is also mandatory to adjust education and training curricula to fast-moving changes in radiology, and to standardise and harmonise training.

Current ESR activities include the European Training Curriculum, the European Training Assessment Programme and the European Diploma in Radiology.

### Big data

So called “big data” is a great opportunity for radiologists to improve quality patient care and may also provide enormous research potential. Currently, hindrances for such adoption include poor quality data, problems with the management of large volumes of data, patient confidentiality, security and lack of interoperability in healthcare systems. Imaging biobanks are an obvious way forward, allowing the accumulation of databases of radiological images and associated biomarkers into a repository. However, current bottlenecks include a lack of infrastructure, standardisation, interoperability and anonymization of data within these biobanks. The ESR Research Committee is working on initiatives such as the Biobanking and BioMolecular resources Research Infrastructure (BBMRI-ERIC) at the European Union (EU) level to establish proof of concept that DICOM data can be integrated into existing biobanks.

### Health technology assessment

Innovations in healthcare come along all the time and carrying out health technology assessment (HTA) has become important in deciding what should be incorporated into clinical practice. Stakeholders involved in decision-making increasingly want to base their judgements on unbiased assessment. The 2011 EU cross-border healthcare directive notably mentions that from 2015, innovations in any area of healthcare should go through HTA^7^.

Radiologists may consult the reports from the Institute of Medicine, the Radiological Society of North America (RSNA) or HTA agencies for guidance. Facilities can also conduct their own mini HTA by combining global evidence with local information.

#### Global conclusions and recommendations

Among the many tasks of professional societies, advocacy, awareness, fostering of integration of quality and safety policies is crucial for our profession. Communication with stakeholders and contribution to the development of a strategic research agenda on radiation protection were singled out.

Key performance indicators are necessary to document quality performance and safe radiology practices. Different approaches for justification could come from a number of organizations implementing guidelines and decision support tools at the point of care.

To improve quality and safety in imaging, tools for justification, optimisation, work quantification and communication with the patient should be available. Customer care should be the basis for tools development and obtaining feedback from stakeholders is essential. Clinical decision support systems (CDSSs) are important to communicate with regulators but also families, patients and the healthcare community. More e-learning tools are necessary.

## Footnotes

1 Survey report submitted for publication in Insights Into Imaging (March 2016)

2 The radiology report as seen by radiologists and referring clinicians: results of the COVER and ROVER surveys,

3 Structured reporting: if, why, when, how and at what expense? Results of a focus group meeting of radiology professionals from eight countries.

4 Radiology Liability Update: Review of Claims, Trends, High-Risk Conditions and Practices, and Tort Reform Alternatives,

5 Medical Malpractice: The Experience in Italy,

6 Liability claims and costs before and after implementation of a medical error disclosure program,

7 Directive 2011/24/EU of the European Parliament and of the Council of 9 March 2011 on the application of patients’ rights in cross-border healthcare http://eur-lex.europa.eu/legal-content/EN/TXT/?uri=CELEX:32011L0024
